# Does community mobilisation improve awareness, approval and uptake of family planning methods among women of reproductive age in Ebonyi State? Experience from a quasi-experimental study

**DOI:** 10.11604/pamj.2019.33.17.17401

**Published:** 2019-05-10

**Authors:** Ifeyinwa Chizoba Akamike, Ijeoma Nina Okedo-Alex, Ugochukwu Chinyem Madubueze, Chukwuma David Umeokonkwo

**Affiliations:** 1 Community Medicine Department, Federal Teaching Hospital, Abakaliki, Ebonyi State, Nigeria

**Keywords:** Community mobilisation, family planning, awareness, approval, uptake, reproductive age

## Abstract

**Introduction:**

family planning programmes have helped in increasing the prevalence of contraceptive use and reducing total fertility rate in developing countries from six to three births per woman. However, its uptake is lower in the rural areas compared to urban areas. This study seeks to elucidate the effect of community mobilisation on awareness, approval and use of family planning among women of reproductive age in the rural areas of Ebonyi state, Nigeria.

**Methods:**

we conducted a quasi-experimental study among women aged 15 to 49 years in two rural communities in Ebonyi state. Using simple random method, we recruited 484 women for the study. We used pre-tested interviewer-administered questionnaire to collect information from the participants. Community awareness and distribution of information, education and communication materials were carried out within one month. We estimated the effect of the intervention on the level of awareness, approval and uptake of family planning methods.

**Results:**

level of awareness increased by 19% (p<0.001) while uptake of family planning increased by 16.7% (p<0.001) in the intervention group. The approval rate was higher in the intervention group compared to the control group (p=0.008). The most commonly used method of family planning was the natural method in intervention and control arms.

**Conclusion:**

although uptake of family planning increased significantly in the study population, the rate is generally low. Given the critical role of the community in family planning programmes, community mobilisation may be deployed to increase uptake of family planning in similar rural communities.

## Introduction

Family planning is an essential component of primary health care and reproductive health [1, 2] and has contributed in improving reproductive health [3]. The objectives of family planning services include encouraging couples to take responsible decisions about pregnancy and enabling them achieve their desires as regards preventing unwanted pregnancy, limiting family size and promoting responsible parenthood, controlling the population and improving the quality of life of the people [1, 4]. Although there are many benefits of family planning and about 225 million women living in developing countries desire to delay pregnancy or stop childbearing, use of any method of family planning has been found to be very low [5, 6]. Globally, modern contraceptive use rose slightly from 54% in 1990 to 57.4% in 2014 and in Africa, it rose from 23.6% to 27.6% [5]. In sub-Saharan Africa, the proportion of married women using a modern method of family planning is less than 20% [7]. In Nigeria, in spite of the high level of knowledge of family planning methods, [8, 9] uptake of modern methods is still low with only 9.7% of married women using a modern method of family planning according to the 2008 national demographic health survey and 9.4% according to 2013 national demographic health survey [8, 10]. This shows a slight decrease in the use of modern family planning methods in the country. Millions of mistimed or unwanted pregnancies occur worldwide every day and this contributes to high rates of induced abortions, maternal morbidity and mortality and infant mortality [11]. In developed countries, almost all married women make use of modern methods of family planning at one point or the other in their reproductive lives but the proportion reporting such use is very low in developing countries [12].

Use of modern methods of family planning is still low in some of the world's poorest and most populous countries. In Africa, poverty, poor access to services and commodities, conflicts, poor coordination of family planning programs and unstable donor funding have contributed in hindering the provision of family planning services [2]. Other factors such as traditional beliefs, religious barriers, and lack of male involvement have also impacted negatively on family planning interventions leading to high rates of unintended pregnancies in many countries [2, 7, 12]. Although family planning services have been made available in various health facilities, these services are not being accessed by women particularly those in the rural areas. Studies have shown that contraceptive prevalence is higher among women in the urban areas when compared to women in the rural areas [9, 13]. Several studies have shown that there is high level of awareness and approval of family planning among women, however, this does not translate to practice of family planning [9, 14-18]. In a study carried out in Ebonyi state among market women of reproductive age, knowledge of contraceptives was high (83.3%) but only 28.3% used a method of family planning [12]. About 70% of the Ebonyi people live in the rural area and the majority of these rural dwellers are farmers. The people therefore believe that having many children will increase the workforce needed for farm work. This has contributed to high fertility rate and low uptake of family planning in the state. Community mobilisation has been shown to have positive impact on acceptance and uptake of health programmes and interventions including family planning [7]. This is because it is able to address the socio cultural barriers to health seeking [19]. Community mobilisation aims to arouse the interests of individuals and groups in the community towards issues that concern them and encourage them to participate actively in finding solutions to their problems [19, 20]. The study was undertaken to determine the effect of community mobilisation on awareness, approval and use of family planning methods among women of reproductive age and provides information on the role of community driven strategies in improving knowledge, approval and use of family planning methods.

## Methods

The study was conducted in two rural communities in Ebonyi state, Nigeria (Igbeagu in Izzi LGA and Ezzamgbo in Ohaukwu LGA). These two communities have comparable levels of trade and commerce, health care services and similar occupations. Rural communities were selected for this study because past demographic surveys have shown that women in urban areas are more likely to accept and practice family planning compared to those in rural areas [8, 10]. In addition, most programs are implemented in urban areas. The study population comprised of women of reproductive age group i.e. 15-49 years, who are married or in a union and who are permanent residents of the study areas. Pregnant women and women who had achieved menopause, but in the reproductive age group were excluded.

**Study design:** the study was a quasi-experimental study comprising of an intervention arm and a control arm and was conducted in three phases: baseline survey, intervention, and three months post intervention surveys.

**Baseline survey:** baseline data from intervention and control groups were collected using pre-tested semi-structured interviewer administered questionnaires to collect information on uptake of family planning methods.

**Intervention:** the intervention for this study was community mobilisation which included the following activities: community awareness campaign; distribution of information, education communication (IEC) materials. The community awareness was preceded by an advocacy visit to the community leader in charge of Igbeagu community. Advocacy was also carried out to some other leaders in the community including the market leader, and some women group leaders in churches. The community awareness campaign was carried out to enlighten community members on the availability of family planning services in the primary health care facilities and other service providers in their community. The awareness campaign was carried out in collaboration with the community leaders. This served as a platform for community leaders and representatives to build community support and following for family planning. The community awareness was carried out on two separate days. The first was carried out at the market square of the community on their major market day, since most of the women usually visit the market on that day. The second was carried out at the village square. Each session lasted for about an hour with an average of 250 people in attendance on each day. Health talks and demonstrations were used for the community awareness. Information, education, communication (IEC) materials were then distributed. Leaflets were distributed to men and women in the community. The leaflets contained pictures conveying information on family planning. The wordings on the leaflets were in English and also the local dialect. The IEC materials were adapted from family planning posters on the internet and modified. The intervention was carried out within one month.

**Post-intervention survey:** three months after the intervention, the same questionnaire that was used for baseline data collection was administered to the same respondents that were surveyed pre-intervention in both intervention and control groups to determine the effect of the intervention on uptake and patterns of use of modern methods of family planning.

**Sample size and sampling technique:** the level of significance and the power of the study were set at 5% and 80% respectively. A minimum sample size of 194 was calculated per study arm. To account for an attrition rate of 20%, the sample size was adjusted to 242. Therefore, a total of 484 women of reproductive age were approached to participate in the survey. Respondents were selected from one community each from Izzi LGA which served as the intervention site and from Ohaukwu LGA as the control site. The list of communities for each LGA was obtained from the LGA headquarters. Igbeagu community was selected from the intervention LGA while Ezzamgbo was selected from the control LGA. Simple random sampling method was then used to select four enumeration areas from each community. All households in the selected enumeration areas were sampled and all eligible women who gave consent were surveyed.

**Data collection:** a pre-tested, semi structured interviewer administered questionnaire was used to collect information from respondents. Information collected include socio-demographic characteristics of the respondents such as age, marital status, level of education and spouse's level of education; awareness of family planning, approval and uptake of family planning methods. The questionnaire was designed and modified from previous studies [18, 21-24]. It was pre-tested among women in another census enumeration area, outside those of the study population. Data was collected over a period of one month by four trained research assistants.

**Data analysis:** data analysis was carried out using Statistical Package for Social Sciences (IBM-SPSS) for Microsoft Window version 20 software. Frequencies and proportions were calculated for socio-demographic characteristics. These were compared between the intervention and control group. The mean age of the respondents in both intervention and control groups were compared with t test difference of two means at 5% level of significance. We calculated the proportion of women who were aware of, approved and used family planning in each group. The proportion was then compared within each group before and after the intervention with chi-square test at 5% level of significance.

**Ethical considerations:** ethical clearance for this study was obtained from Research and Ethics Committee of Federal Teaching Hospital Abakaliki, Ebonyi State of Nigeria. Written informed consent was obtained and confidentiality ensured.

## Results

Table 1 shows that the respondents in both groups were comparable in their socio-demographic characteristics. The mean age of respondents in the intervention arm was 29.39±6.1 while that for the control arm was 28.36±5.9. Majority of the respondents and their spouses had at least secondary education in both intervention and control arms. Majority of the respondents had been counselled on family planning (intervention: 76.4%; control: 88.0%) and the main sources of family planning counselling in the both communities were health facilities, health workers and churches. Natural method of family planning was the preferred choice in both the intervention (56.4%) and control group (58.3%). The major reasons for approving family planning were child spacing, preventing unwanted pregnancy and limiting family size (Table 2). Table 3 shows that awareness of family planning was 68.2% before intervention in the intervention arm with a statistically significant increase in awareness after the intervention. Uptake of family planning was low at 22.2% before the intervention. There was however a 16.7% increase in uptake three months post-intervention in the intervention arm, this increase in uptake was also statistically significant. Majority of the respondents approved of family planning before the intervention (90%), which increased to 97% after the intervention. The increase in proportion of respondents that approved of family planning was also statistically significant. Table 4 shows that awareness and approval were both high in the control arm, however there was no statistically significant increase in awareness and uptake at the end of the study. Uptake of family planning was low in the control arm at the beginning of the study. Though there was a 7.4% increase in uptake, this increase was not statistically significant. The study also shows that the most common methods of family planning known by respondents were the injectables and the pills, followed by the natural method and then the barrier method in the intervention arm. In the control arm, the commonest method known was the injectable, followed by the barrier method, then the pills and natural method. Other methods known include the implant, intrauterine contraceptive device (IUCD) and surgical method. The natural method was the most common method of family planning being used by the respondents in both arms (over 50% of the respondents). The IUCD and the surgical methods were not being used by any of the respondents in both arms.

**Table 1:** socio-demographic characteristics of respondents in intervention and control arms

Variable	Intervention n (%)	Control n (%)	*χ^2^*	*p value*
**Age Group(years)**				
15-19	4(1.7)	7(2.9)		
20-29	121(50.0)	132(54.6)		
30-39	102(42.1)	91(37.6)		
40-49	15(6.2)	12(4.9)		
Mean age (mean ±SD)	29.39±6.1	28.36±5.9	1.873**	0.06
**Marital status**				
Married	236(97.5)	239(98.8)	FT	0.50
Cohabiting	6(2.5)	3(1.2)		
**Level of education**				
No Education	5(2.0)	6(2.5)		
Primary education	41(17.0)	34(14.1)	2.2	0.5
Secondary education	147(60.7)	141(58.2)		
Tertiary education	49(20.3)	61(25.2)		
**Educational level of husband**				
No education	10 (4.1)	7 (2.9)	6.0	0.1
Primary Education	53 (21.9)	34 (14.1)		
Secondary Education	104 (43.0)	116 (47.9)		
Tertiary Education	75(31.0)	85 (35.1)		

** T-test FT=Fischer Exact test

**Table 2:** baseline distribution of the family planning use, approval and counseling among the intervention and control groups

Variable	Intervention	Control
**Method of Family Planning Used**		
Natural method	31 (56.4)	42 (58.3)
Modern method	24 (46.6)	30 (41.7)
**Reasons for Approval of Family Planning**		
Child spacing	176 (72.2)	179 (74)
Prevents unwanted pregnancy	134 (55.4)	149 (61.6)
Limits family size	168 (69.4)	156 (64.5)
Improves mother’s health	21 (8.7)	18 (7.4)
Improves child’s health	5 (2.1)	8 (3.3)
Counselled for family planning		
Yes	185 (76.4)	213 (88.0)
No	57 (23.6)	29 (12.0%)
**Source of counselling for family planning**		
Health facility	161 (66.5)	185 (76.4)
Health worker	40 (16.5)	39 (16.1)
Church	35 (14.5)	23 (9.4)
Radio/Television	1 (0.4)	11 (4.5)

**Table 3:** within group comparison of awareness, approval and uptake of family planning in the intervention arm

Variable	Before	After	*χ^2^*(*p value*)
**Awareness of family planning**			
Yes	165(68.2)	177(87.2)	23.5(<0.001*)
No	77(31.8)	26(12.8)	
**Uptake of family planning**			
Yes	55(22.7)	80(39.4)	14.8(<0.001*)
No	187(77.3)	123(60.6)	
**Approval of family planning**			
Yes	220(90.9)	196(97.0)	7.0(0.008*)
No	22(9.1)	6(3.0)	

* Statistical significance

**Table 4:** within group comparison of awareness, approval and uptake of family planning in the control arm

Variable	Before	After	*χ^2^*(*p value*)
**Awareness of family planning**			
Yes	217(89.7)	190(91.8)	0.8(0.4)
No	25(10.3)	17(8.2)	
**Uptake of family planning**			
Yes	72(29.8)	77(37.2)	2.9(0.09)
No	170(70.2)	130(62.8)	
**Approval of family planning**			
Yes	215(88.8)	184(88.9)	0.0(1.0)
No	27(11.2)	23(11.1)	

## Discussion

Our study has demonstrated the level of awareness, approval and uptake of family planning methods. The study has also shown the effectiveness of community-based intervention in improving the use of family planning methods by married women. The mean age of respondents in the intervention arm was 29.39±6.1 while that for the control arm was 28.36±5.9. Majority of the respondents and their spouses had at least secondary education in both intervention and control arms. Majority of the respondents were aware of family planning and majority also approved of family planning in both intervention and control groups. This corroborates the findings of previous studies [9, 14-18]. This high level of awareness and approval may be explained by the fact that there has been a lot of education about family planning especially in antenatal and immunisation clinics in recent times. Despite the high levels of awareness and approval, uptake of family planning was found to be low in both the intervention and control arms (22.7% and 29.8% respectively). This finding also agrees with previous studies [12, 25-27]. The most commonly used method of family planning in both arms was the natural method despite the fact that the most commonly known methods were the injectable, pills and barrier methods. This observed pattern of preference for natural method could be because the natural method has no side effects and does not require one to visit the hospital or to take any form of medications. The study further revealed that none of the respondents was using IUCD or the surgical method of family planning, probably because the services are not offered in any of the health facilities serving the communities that were surveyed.

The community mobilisation interventions implemented in this study resulted in statistically significant increase in uptake, awareness and approval of family planning methods. At post intervention, uptake of modern methods of family planning increased by 16.7% in the intervention arm (p<0.001). There was also statistically significant increase in awareness and approval of family planning in the intervention arm. There was no statistically significant difference in the control arm. Community mobilisation has been reported to increase perception and uptake of reproductive health services including family planning [19, 28-32]. Each strategy of community mobilisation implemented in this study had a unique effect on addressing community-related barriers to uptake of family planning. Firstly, the community awareness campaign which was implemented with the support of community leaders in the community generated some level of consciousness in the community about family planning. Community awareness campaigns have been used in health promotion for improving demand for maternal and child health services, malaria control interventions and HIV prevention strategies [33, 34]. Secondly, distribution of IEC materials created more consciousness and provided additional information on benefits of family planning. The pictorial presentation of the information made it relatable and easier to understand. An added benefit of the IEC material is that it enables reinforcement of information. A similar finding was reported in a study that explored the use of behavior change communication to address perceptions of postpartum return to fecundity and contraceptive adoption [35]. This study is not without limitations. Firstly, the study was carried out among women in two rural areas, one as intervention and the other as control and so the finding may not be generalised to other populations. Secondly, the study was a quasi-experimental study without randomisation. This could also have led to biases in the study.

## Conclusion

Although uptake of family planning increased significantly in the study population, the rate is generally low. Given the critical role of the community in family planning programs, community mobilisation may be deployed to increase uptake of family planning in similar rural communities. However, further studies in larger populations need to be carried out to identify more effective strategies of improving uptake. More work needs to be done by community health workers in the area of educating the women and the entire community on the benefits of family planning.

### What is known about this topic

- Awareness of family planning is high among women of reproductive age;
- Uptake of family planning is low particularly in rural areas;
- There is high level of approval of family planning among women of reproductive age.

### What this study adds

- Community based strategies are useful in improving awareness, approval and uptake of family planning methods;
- Although uptake improved, it is still generally low and therefore calls for further research.

## Competing interests

The authors declare no competing interests.

## References

[1] Obionu C (2007). Primary Health Care for Developing Countries.

[2] WHO (2008). Repositioning family planning: Guidelines for advocacy action.

[3] Fusi-Ngwa C, Payne V, Asakizi A, Katte B (2013). Knowledge and practice of family planning in Dschang municipality, Cameroon. African Journal of Reproductive Health.

[4] Ikechebelu JI, Joe-Ikechebelu NN, Obiajulu FN (2005). Knowledge, attitude and practice of family planning among Igbo women of south-eastern Nigeria. J Obstet Gynaecol.

[5] World Health Organisation (2016). Family planning/contraception factsheet.

[6] Pack AP, McCarraher DR, Chen M, Okigbo CC, Albert LM, Wambugu S (2014). Factors associated with unmet need for modern contraception in post-conflict Liberia. Afr J Reprod Health.

[7] Chigbu B, Onwere S, Feyi-Waboso P, Kamanu C, Aluka C, Ezirim O (2014). The impact of collaboration and family planning counseling in the community setting. Journal of Medical Investigation and Practice, ABSU-MRS & College of Medicine and Health Sciences.

[8] National Population Commission, ICF International (2013). Nigeria Demographic And Health Survey 2013.

[9] Oye-Adeniran BA, Adewole IF, Umoh A, Oladokun A, Gbadegesin A, Ekanem EE (2006). Community-based study of contraceptive behaviour in Nigeria. Afr J Reprod Health.

[10] National Population Commission (2008). Nigerian Demographic And Health Survey.

[11] Mwaikambo L, Speizer IS, Schurmann A, Morgan G, Fikree F (2011). What works in family planning interventions: a systematic review. Stud Fam Plann.

[12] Egede JO, Onoh RC, Umeora OUJ, Iyoke CA, Dimejesi IBO, Lawani LO (2015). Contraceptive prevalence and preference in a cohort of south-east Nigerian women. Patient Prefer Adherence.

[13] Decker M, Constantine NA (2011). Factors associated with contraceptive use in Angola. Afr J Reprod Health.

[14] Aryeetey R, Kotoh AM, Hindin MJ (2010). Knowledge, perceptions and ever use of modern contraception among women in the Ga East District, Ghana. Afr J Reprod Health.

[15] Teye JK (2013). Modern contraceptive use among women in the Asuogyaman district of Ghana: is reliability more important than health concerns. Afr J Reprod Health.

[16] Ochako R, Mbondo M, Aloo S, Kaimenyi S, Thompson R, Temmerman M (2015). Barriers to modern contraceptive methods uptake among young women in Kenya: a qualitative study. BMC Public Health.

[17] Avong H (2000). Perception of and Attitude toward the Nigerian Federal Population Policy, Family Planning Program and Family Planning in Kaduna State, Nigeria. Afr J Reprod Health.

[18] Onwuzuruike B, Uzochukwu B (2001). Knowledge, attitude and practice of family planning amongst women in a high density low income urban of Enugu Nigeria. Afr J Reprod Health.

[19] United States Agency for International Development (2006). Community Mobilisation: Improving Reproductive Health Outcome.

[20] Olise P (2012). Primary Health Care for Sustainable Development. 2.

[21] Gertrude N (2013). Missed opportunities for modern family planning services among women attending child health clinics in Iganga/ Mayuge Demographic Surveillance site.

[22] Nyambe N (2012). The impact of peer education on family planning uptake in Lusaka HIV Clinics: a gender perspective.

[23] Wendo BM Barriers to uptake of long term and permanent family planning methods among HIV infected postpartum mothers in Kenyatta National Hospital.

[24] Abdulrazaq AG, Kabir S, Mohammad NS, Suleiman IH (2014). The effect of educational intervention on family planning knowledge, attitudes, and practices among married women in a military barrack in northern Nigeria. Afr J Reprod Health.

[25] Asekun-Olarinmoye EO, Adebimpe W, Bamidele J, Odu O, Asekun-Olarinmoye I, Ojofeitimi EO (2013). Barriers to use of modern contraceptives among women in an inner city area of Osogbo metropolis, Osun State, Nigeria. Int J Womens Health.

[26] Etokidem AJ, Ndifon W, Etowa J, Asuquo EF (2017). Family planning practices of rural community dwellers in Cross River State, Nigeria. Niger J Clin Pract.

[27] Idris S, Sambo M, Ibrahim M (2013). Barriers to utilisation of maternal health services in a semi-urban community in northern Nigeria: the clients? perspective. Niger Med J.

[28] Adongo PB, Tapsoba P, Phillips JF, Tabong PT, Stone A, Kuffour E (2013). The role of community-based health planning and services strategy in involving males in the provision of family planning services: a qualitative study in Southern Ghana. Reprod Health.

[29] Malwenna L, Jayawardana PL, Balasuriya A (2012). Effectiveness of a community based health educational intervention in reducing unmet need for modern methods of family planning among ever married reproductive age women in the Kalutara district, Sri Lanka. Int J Collab Res Intern Med Public Heal.

[30] Pathfinder International (2006). Improving reproductive health through community-based services: 25 years of Pathfinder international experience.

[31] Tawye Y, Jotie F, Shigu T, Ngom P, Maggwa N (2005). The potential impact of community-based distribution programmes on contraceptive uptake in resource-poor settings: evidence from Ethiopia. Afr J Reprod Health.

[32] Uneke CJ, Ndukwe CD, Ezeoha AA, Chukwuemeka H (2014). Policy brief improving maternal and child healthcare programme using community-participatory interventions in Ebonyi State Nigeria. Int J Heal Policy Manag.

[33] Marston C, Renedo A, Mcgowan CR, Portela A (2013). Effects of community participation on improving uptake of skilled care for maternal and newborn health: a systematic review. PLoS One.

[34] Shumba CS, Atuhaire L, Memiah P, Atukunda R (2013). Assessment of community mobilization and home-based HIV counselling and testing offered by health facilities in rural Uganda. Afr J Reprod Health.

[35] Cooper CM, Ahmed S, Winch PJ, Pfitzer A, Mckaig C, Baqui AH (2014). Patient education and counseling findings from the use of a narrative story and leaflet to influence shifts along the behavior change continuum toward postpartum contraceptive uptake in Sylhet District, Bangladesh. Patient Educ Couns.

